# Under-triage in telephone consultation is related to non-normative symptom description and interpersonal communication: a mixed methods study

**DOI:** 10.1186/s13049-017-0390-0

**Published:** 2017-05-15

**Authors:** Hejdi Gamst-Jensen, Freddy K. Lippert, Ingrid Egerod

**Affiliations:** 1 Emergency Medical Services Copenhagen, University of Copenhagen, Telegrafvej 5, 2. Sal, 2750, Ballerup, Denmark; 2 Trauma Centre, Rigshospitalet, University of Copenhagen, Blegdamsvej 9, 2100, Copenhagen, Denmark

**Keywords:** Telephone consultation, ICD codes, Out-of-hours medical care, Prehospital acute medicine, Telephone hotline, Triage (under-triage)

## Abstract

**Background:**

Telephone consultation and triage are used to limit the workload on emergency departments. Lack of visual cues and clinical tests put telephone consultations to a disadvantage compared to face-to-face consultations increasing the risk of under-triage. Under-triage occurs in telephone triage; however why under-triage happens is not explored yet. The aim of the study was to describe situations of under-triage in context, to assess the quality of under-triaged calls, and to identify communication patterns contributing to under-triage in a regional OOH service in the capital region of Denmark.

**Methods:**

Explanatory simultaneous mixed method with thematic analysis and descriptive statistics was chosen. The study was carried out in an Out-Of-Hours service (OOH) in the Capital Region of Denmark, Copenhagen. Under-triage was defined as Potentially Under-Triaged Calls (PUTC) by specific criteria to an OOH Hotline, and identification by integration of three databases: Medical Hotline database, Emergency number database, including the Ambulance database, and electronic patient records. Distribution of PUTC were carried out using ICD-10 codes to identify diagnosis and main themes identified by qualitative analysis of audio recorded under-triaged calls. Study period was October 15^th^ to November 30^th^ 2014.

**Results:**

Three hundred twenty seven PUTC were identified, representing 0.04% of all calls (*n* = 937.056) to the OOH. Distribution of PUTC according to diagnoses was: digestive (24%), circulatory (19%), respiratory (15%) and all others (42%). Thematic analysis of the voice logs suggested that inadequate communication and non-normative symptom description contributed to under-triage.

**Discussion:**

The incidence of potentially under-triage is low (0.04%). However, the over-representation of digestive, circulatory, and respiratory diagnoses might suggest that under-triage is related to inadequate symptom description. We recommend that caller and call-handler collaborate systematically on problem identification and negotiate non-normative symptom description.

**Conclusion:**

The incidence of under-triage is low (0.04%). However, the over-representation of digestive, circulatory, and respiratory diagnoses might suggest that under-triage is related to inadequate symptom description. We recommend that caller and call-handler collaborate systematically on problem identification and negotiate non-normative symptom description.

## Background

Telephone consultation are often chosen over face-to-face consultations in Out Of Hours (OOH) services to ensure cost effectiveness [1], however telephone consultations are associated with uncertainty due to the lack of visual cues [2, 3]. The accuracy of telephone triage has been shown to vary from 49 to 98% [4], suggesting that the issue might affect patient safety. The decision-making process in telephone consultations is influenced by call-handler’s dual task of offering advice (helping the caller) while also acting as gatekeeper [5]. Thus, more knowledge about the pattern of communication that might precipitate under-triage could be used to improve the call-handler/caller interaction. This study aims to describe situations of under-triage in context, to assess the quality of under-triaged calls, and to identify communication patterns contributing to under-triage in a regional OOH service in the capital of Denmark.

## Methods

**Fig. 1:**
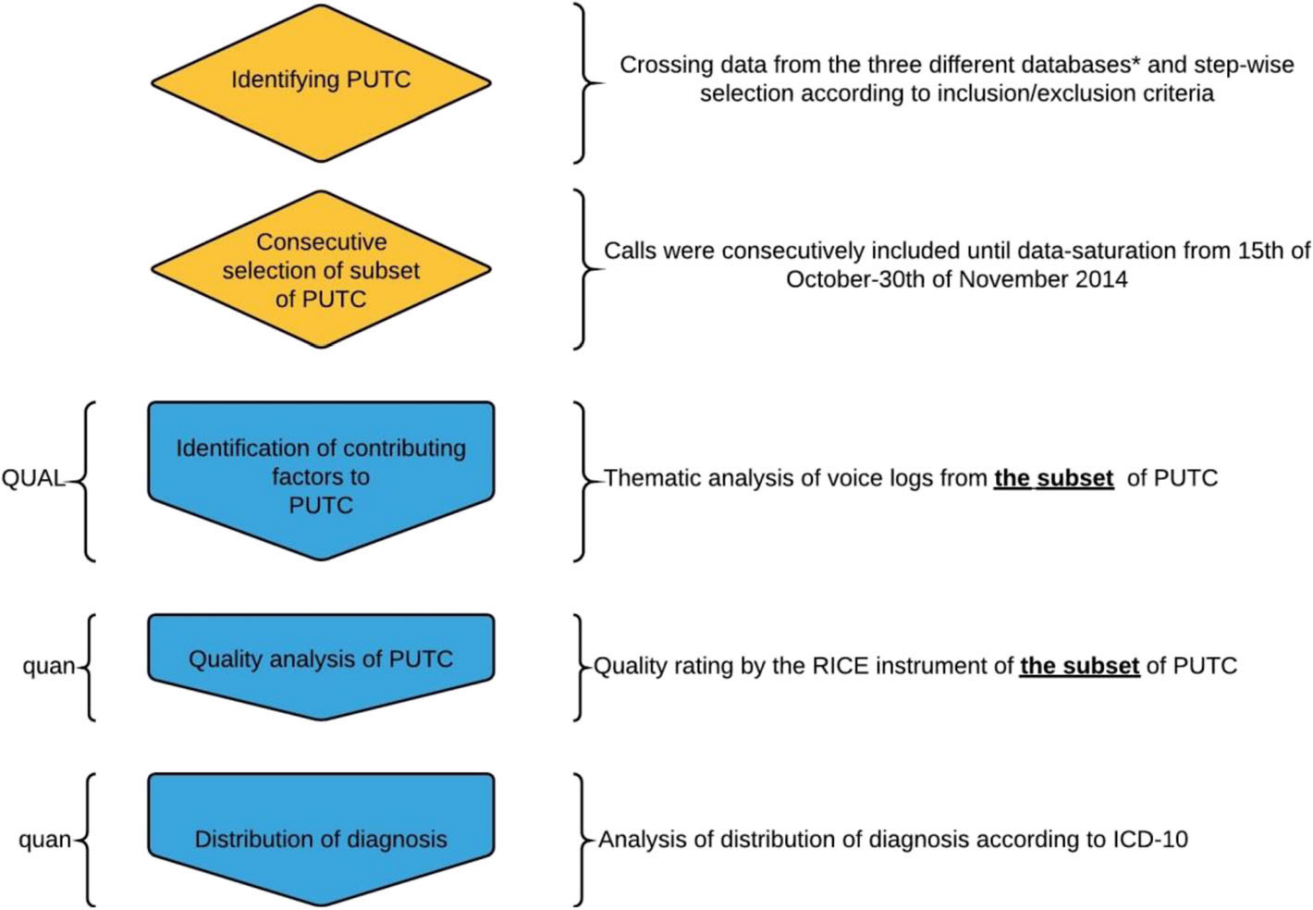
Overview of methods, priority and timing of the quantitative and qualitative strand. Identification of Potentially Under-Triaged Calls were conducted by crossing data from medical hotline 1813, emergency hotline 112, and the electronic patient record “Opus”. PUTC = Potentially Under-Triaged Calls

The study had an explanatory simultaneous mixed methods design involving two retrospective datasets [6]. Data consisted of a quantitative strand and a qualitative strand. The qualitative strand was weighted more than the quantitative strand (Fig. 1).

### Setting

The study used data from the OOH service in the Capital Region of Denmark, Copenhagen (hereafter Copenhagen). In this region the OOH is integrated in the Emergency Medical Services and covers a population of 1.7 million citizens (callers) who can contact the OOH Medical Hotline and will be triaged to receive a home visit, s*cheduled* for a consultation at an emergency department or acute care clinic, hospitalised or given advice on self-care, or advised to see their usual GP. The call-handler responding to the call is either a nurse or a physician. Triage and determination of urgency is guided by an electronic decision tool based on the symptoms described by the caller. The triage tool is a detailed guideline to support decision making. The guideline is for nurses a strict protocol where deviations have to be approved by a physician. For physicians the triage tool is a guideline that can be deviated from, however this should be documented. The decision tool is divided into three main sections: somatic illness, somatic injury and psychiatric illness. Emergency calls for potential life-threatening symptoms or injury and request for an ambulance are handled through a different telephone number, 112.

### The quantitative study

**Table:** 

Data in the quantitative strand were used to identify Potentially Under-Triaged Calls (PUTCs) and to describe the distribution of diagnoses using the ICD-10 codes. For a call to be identified as a PUTC it had to meet three criteria: **BOX: PUTC criteria** 1) Caller used the Medical Hotline number, and was NOT offered a home visit and was NOT offered an appointment at an Emergency Department, or hospitalised, **AND** 2) Caller used the Emergency number 112 for an ambulance within two hours of the first contact, **AND** 3) Caller was admitted to hospital later by ambulance.	

Data were retrieved from administrative databases “Medical Hotline 1813” (the OOH call system), Emergency Number 112 (the ambulance database), and the electronic patient record used in the regions hospitals (using the ICD-10 code for admission). Inclusion: calls identified as PUTC. Exclusion: calls concerning psychiatric complaints, calls unrelated to the initial somatic complaints (e.g. calls regarding logistics), calls concerning febrile seizures in children.

Data were described calculating percentage of ICD-10 codes and grouping these into sense making clusters.

### The qualitative study

The specific aim of the qualitative strand was to identify contributing factors to PUTC, using thematic analysis of audio recorded voice logs.

#### Data generation

The qualitative sample was a subset of calls (voice logs) to the Medical Hotline 1813 selected by consecutive criterion sampling from October 15^th^ to November 30^th^, 2014 until data saturation by information redundancy. The criterion sample consisted of calls identified as PUTC in the quantitative strand.

#### Data analysis

The voice logs were retrieved from an internal database and transcribed verbatim. Initial deductive coding was performed using the four components of the RICE scale [7, 8] to structure the data corpus. The Rice scale is a Dutch assessment tool of call-handlers’ communication skills. The items in the rating scale are structured in accordance with the sequence in telephone triage calls: Reason for calling, Information gathering, Conclusion and Evaluation (RICE) which are sub-divided into 17 items. Inductive thematic analysis was carried out according to the active participant (call-handler vs. caller). The initial codes were clustered into themes, data were systematically reviewed to ensure that name, definition and exhaustive set of data supported the theme. It was verified that the results were representative of the collected calls by re-reading the transcripts and re-assessing the themes. The results were researcher triangulated and discrepancy solved by discussion and consensus [9].

To rule out that under-triage was the result of poor quality communication a quality assessment was made with the RICE instrument, poor quality (RICE < 80%) and good quality (RICE >80%). This criterion was provided by the author of the RICE criteria (personal correspondence).

## Results

### Quantitative results

**Fig. 2:**
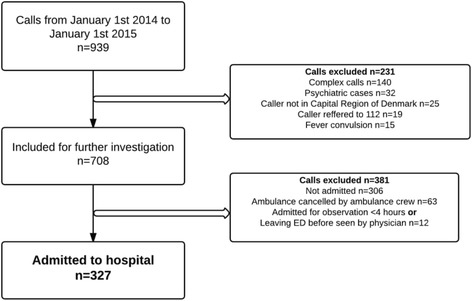
Flowchart of cases included

The Medical Hotline 1813 received 937 056 calls in 2014 of which approximately 40% were triaged to self-care or GP. Applying the PUTC criterion meant that a total of *n* = 939 calls were eligible for the study, Fig. 2.

**Fig. 3:**
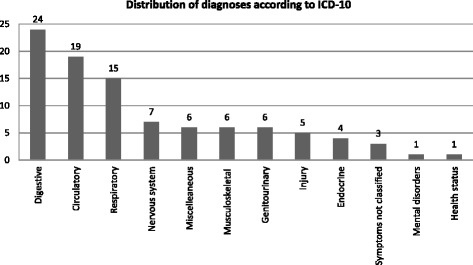
Distribution of PUTC according to diagnosis at admission in ICD-10 codes. *Miscellaneous includes diseases related to pregnancy, childbirth, certain infections, and diseases of eye and skin

From the 939 eligible calls, 612 were excluded according to exclusion criteria, leaving 327 for analysis in the study. As such, approx. 35% of the study population were PUTC and included in the description of distribution of diagnosis according to ICD-10, Fig. 3.

### Qualitative results

We analysed 31 calls regarding 19 patients from the sample of 327 calls to the Medical Hotline 1813. According to the RICE score, 21 of the 31 calls were of acceptable quality, Table 1.

**Table 1:** Demographics of potentially under-triaged calls (PUTC)

	Caller	Patient, Sex/age	OOH hotline problem	Admission diagnosis	RICE rating	Other
1	Patient	Male, 64	Smoke inhalation	Observation of smoke inhalation	71%	Admission for social reasons
2	Patient	Female, 79	Abdominal pain	Observation of AMI	86%	n/a
3	Daughter	Female, 68	Nose bleed	Defect of septum	80% (a)^a^ 77% (b)	n/a
4	Friend	Female, 40	Dizziness	Vertigo	56%	Language difficulties
5	Mother	male < 1	Diarrhea and vomiting	Syncope	89% (a) 82% (b)	n/a
6	Mother	Female, 4	Laryngitis	Epiglottis	90% (a) 79% (b) 87% (c)	n/a
7	Husband	Female, 75 years	Abdominal pain	Missed STEMI	77% (a) 75% (b) 95% (c)	n/a
8	Mother	Female, 8	Diarrhea and vomiting	Dehydration	88%	n/a
9	Patient	Female, 53	Allergic reaction	Perioral edema	84%	n/a
10	Patient	Male, 45	Abdominal pain	Gallstone	94% (a) 84% (b)	n/a
11	Patient	Female, 81	Abdominal pain	Gallstone	89%	n/a
12	Patient	Female, 17	Abdominal pain	Abdominal pain	98% (a) 60% (b)	n/a
13	Patient	Female, 45	Headache/ dizziness	Chronic post-traumatic headache after SAH	96% (a) 82% (b)	Frequent caller
14	Patient	Male, 64	Edema of lower extremity	Obs. DVT	75%	n/a
15	Spouse	Male, 28	Fainting	Observation for neurological problem	88% (a) 92% (b)	n/a
16	Spouse	Male, 25	Hyperglycemia and intoxication	Hyperglycemia	94%	n/a
17	Friend	Male, 56	Fever and abdominal pain	Chronic liver cirrhosis	83% (a) 58% (b)	n/a
18	Patient	Female, 28	Bleeding after vaginal delivery 5 days earlier	Small post-partum bleeding	89% (a) 95% (b)	n/a
19	Daughter	Male, 52	Fever and malaise	Sepsis	29%	n/a

^a^The letter a indicating the first call, b a second call, and c a third call

Inductive coding of the data led to 18 codes related to the call-handler response and 16 codes related to the caller response. The main theme was identified as ‘Co-constructing the problem’ (Table 2).

**Table 2:** Codes and themes derived from complete coding

Participant	Codes	Themes	CO-CONSTRUCTING THE PROBLEM
Call-handler response	Clarifying history of problem Acknowledging caller experience Individualized communication Providing advice in blocks Empowering caller Educating caller Agreement of plan	The professional communication	
	Condescending, irritated, patronizing communication Ignoring patient perspective, minimizing magnitude of problem	The non-professional role	
	Lack of control with conversation Providing unclear advice Disagreeing with caller Non-individualized communication Unable to collaborate with caller	The non-professional Communication	
	Gate keeper Fixation error Uncharacteristic problem Lack of agreement on problem, urgency and plan	The difficult decision making	
Caller response	Clear description of problem Clear description of temporal progression of problem Self-evaluation of problem Agreement of plan	The constructive caller	
	Vague and/or lengthy description of actual problem Cerebrally impaired Time line Insecurity Talking down symptoms	The complicated caller	
	Lacking problem solving/self-care before calling Contradictive information (+vocal appearance)	The un-constructive caller	
	Caller under the influence Caller is very emotional	The caller is inebriated/intoxicated	
	Calling during GP office hours Unrealistic expectations	The caller has unrealistic expectations of outcome	
	Caller is not patient, caller is not with patient	Proximity to patient (physical/relative)	

### Call-handler response

#### Professional communication

Most importantly the professional communication would enhance collaboration and target individual needs, as well as empowering and educating the caller. As seen in a consultation with the mother of a child with laryngitis: “*If it gets any worse or if you think you can’t handle it yourself, call for an ambulance*”. The caller was invited to take part in the decision and treatment plan.

#### Non-professional role

The non-professional role was manifest in blocking and discouraging communication presented by the call-handler as expressions of: Irritation, condescending, or patronizing and characterized by a disagreement of magnitude of the problems and distrust in the described problem. Moreover, the decision making process was impaired by a non-objective approach to the caller. An example of this was the call-handler dismissing the caller or the symptom blocking further information collection. *“…and if you call again in 10 min saying that it (the temperature) is 40° Celsius then I won’t believe you”.*

#### Non-professional communication

Factors that contributed to non-professional communication were: *Lack of control of conversation* in the situations where the caller was under the influence of substances/drugs, fast talking, or continuing to repeat questions and answering themselves, or the very emotional caller. A contributing factor regarding the call-handler was providing *unclear advice.* The advice could be overly complex making it difficult for the caller to focus on one or two clear pieces of advice. *Non-individualized communication* was present in a conversation on self-care advice. *(Call-handler) “Do you have any baking soda in the cupboard?” (Caller):” no, what is that?” (call-handler): “The chemical formula is bicarbonate of soda”.* Nothing was achieved by this explanation and the caller was left without advice.

#### Complex decision making

Assessment of urgency and plan were potentially complicated by several factors. Problems unrelated to the illness (e.g. uncertainty of address) or non-normative symptoms (non- textbook symptoms) led to complex decision making. Even during professional communication the call-handler might overestimate the self-care ability of the patient or might assume that the problem would be self-limiting. As an example, an elderly woman called with severe abdominal pain, which she compared to her experience with kidney stones. Her breathing was audibly affected. The call-handler’s response to the patient was: *“Try taking two paracetamol and go to bed and rest a little and see if it doesn’t resolve … it is probably just a stomach infection”.* Later the same patient called for an ambulance and was triaged as chest pain. Complex decision making can be caused by horizontal reasoning, causing the call-handler to fixate on a wrong diagnosis - in this case a stomach infection the call-handler is convinced that the problem is self-limiting and does not evaluate the symptoms and complaints laterally. The last theme; *lack on agreement on problem, urgency and plan* was seen when the caller and call-handler failed to collaborate and the call was terminated without a clear agreement.

### Caller response

#### The constructive caller

*The constructive caller* would provide the call-handler with a clear description of the problem and with clear normative symptoms. However, a timeline to the description of the problem could both be an advantage or a barrier to correct triage, e.g. if the caller judged the problem to be self-limiting. This paradox was represented in the example of a young man who called in with uncontrollable jerking: “*I have had it like I’m getting a sinus condition but it’s just getting worse and worse*. However, in situations when callers minimized the symptoms, the call became complicated and the decision making process compromised. An example was a middle-aged man who called in with severe abdominal pain*: “I don’t know, maybe I’m just constipated or I have eaten too much”*. The patient was subsequently admitted with a gall stone.

#### The complex caller

The urgency-estimation could be complicated by a *lengthy and unclear description of symptoms*. An example was a woman who called complaining of a severe headache. The caller had suffered a non-traumatic *brain injury* and had acquired a chronic headache. Obtaining the medical history was complicated by the caller’s impaired speech as a result of the injury. Initially the caller declined the offer to be seen at the hospital. Half an hour later she called the emergency Medical Hotline for an ambulance. Some callers appeared insecure when they called, e.g.: *“I don’t know if it is a problem that can wait, I just wanted some advice”.* The complicated caller failed to communicate a clear problem to the call-handler, and the caller might even disagree on the plan without saying so.

#### The un-constructive caller

Callers that had not tried to solve their problem on their own received advice from the call-handler (e.g. pain killers, measure temperature) and were asked to call again if the problem did not resolve. In some cases the un-constructive caller could trigger a non-professional response from the call-handler. E.g. a daughter called on behalf of her father who had severe pain passing urine. (*Call-handler): “did you call his doctor?” (Caller):” No, he’s closed for the day”. (Call-handler): “Then they will refer you to another number on the answering machine”; “In any case we are not coming home to your father” (Caller):” But it seems acute, and I’m working within health care so I know that he’s ill”. (Call-handler): “in that case you should know what a normal temperature is* (referring to earlier in the conversation where the caller asked what a normal temperature was).

#### The caller is not in control

If the caller did not have emotional self-control or was cognitively impaired due to intoxication, collaboration with the call-handler was made difficult. A woman called on behalf of a friend who had a problem with cirrhosis and vomiting is an example of the caller *under the influence* of unknown substances. The caller was unable to present a problem, was contradicting herself, and gave a very lengthy and unstructured description of the reason for calling. The call was terminated by the call-handler stating: “*this is not a problem for the Hotline*!” Shortly thereafter the woman called for an emergency ambulance and the patient was admitted until the following day on social reasons.

#### The unrealistic caller

Some callers had an unrealistic expectation to the outcome of the telephone consultation. One young woman called the Hotline with complaints of gastroenteritis. She was very emotional and not able to collaborate with the call-handler. She wanted an ambulance to come and give her some medicine and ended the call when this was refused. Because of her emotional state when she called for an emergency ambulance she was brought to the hospital and admitted until the following day. Another example of unrealistic expectations was calling during GP opening hours. It was seen that this could trigger a negative and non-professional response from the call-handler.

#### The caller’s proximity to patient

Caller proximity to the patient was also seen to contribute to a difficult telephone consultation. This could be due to physical distance, or if the call was made by a proxy who was not closely related to the patient e.g. *“my dad is having pain when urinating, he is cursing and feeling really bad”*, call-handler; “*can I talk to him?”* Caller; *“No, I’m not with him right now; I talked to my mother, who asked me to phone”*. Physical distance where the caller and patient were not at the same location offered a specific barrier to symptom description, and was often resolved with the request to call back when in the presence of the patient.

## Discussion

Thematic analysis of the voice logs suggested that non-normative symptom and poor communication description contributed to under-triage. The study demonstrated a generally low incidence of potentially under-triage calls of 0.04%. The most frequent categories of admission diagnoses were related to digestive, circulatory and respiratory symptoms. The use combining quantitative and qualitative methodology in this study helps to illustrate different perspectives of the complex issue of potentially under-triage in low urgency calls in a Danish setting.

### Strengths and limitations

The study was conducted during the first year after the re-organization of the OOH service in the Capital Region of Denmark, and thereby included new staff and guidelines. This could have affected the results. The definition of PUTC applied strict criteria to the inclusion of calls. This definition might have been too strict to estimate the actual under-triage, but it did illustrate calls with manifest issues of mis-communication. Moreover, the definition did not take into account the cases that did not make a second call for an emergency ambulance within the time limit, or were admitted by their GP, or even died. Furthermore, the *potentially* under-triaged calls did not take into consideration that some outcomes could be due to a natural deterioration of the patient. The quality analysis of the calls was performed using the RICE instrument only validated in the Nederland. Application of the instrument enabled a quality assessment of the calls showing that poor communication was not the sole contributing factor to under-triage.

### Comparison with existing literature

When dealing with low urgency alternative decision making strategies might be used [2], and internal factors related to the call-handler/caller might confound the triage outcome. The large proportion of abdominal problems in the PUTC, underlines the issue with non-normative symptom description. Abdominal pain is considered to be difficult to evaluate even with lab tests and even when the clinical symptoms are moderately manifest [10], because of many possible differential diagnoses. It is recommended to use clinical decision tools for diagnosis to minimize false positive operative procedures [11]. The medical diagnosis is considered monologic and not dialogic [12], but in absence of the lab tests and clinical observations the co-construction of the problem demands dialog [13], which makes the need for good quality communication even greater. In the present study it was made clear that certain caller characteristics could trigger the call- handler to an un-constructive response. This was seen in the calls where the caller was intoxicated or had unrealistic expectations to the outcome of the call. The stereotype of the intoxicated caller, the caller with limited language proficiency or the un-constructive caller can confound the decision-making process, because the voice utterance is a personal signature [12]. One of the factors that could explain potentially under-triage could be fixation error. Fixation error as term is widely used in emergency medicine for situations where the health professional solely concentrates upon one single aspect of a case to the detriment of other more relevant aspects [14]. There is no simple solution for fixation errors but the introduction of lateral thinking and closed loop communication could alleviate some of the problems and reduce inappropriate gate-keeping [15, 16].

### Implications for research and/or practice

The construction of a scale for the callers’ subjective perception of the situation, like the pain scale, could prove valuable in telephone triage. Such a scale could incorporate the callers’ perspective and thereby gaining more information than a non-normative symptom description and bypassing some of the internal factors related to the call-handler.

## Conclusion

The incidence of potentially under-triage is low (0.04%). However, the over-representation of digestive, circulatory, and respiratory diagnoses might suggest that under-triage is related to inadequate symptom description. We recommend that caller and call-handler collaborate systematically on problem identification and negotiate non-normative symptom description.

## Abbreviations

GP: General practioner
OOH: Out-of-hours services
PUTC: Potentially under-triaged calls
